# Autoimmune Autonomic Ganglionopathy Presenting as Constipation

**DOI:** 10.7759/cureus.22108

**Published:** 2022-02-10

**Authors:** Abdelwahab Ahmed, Shannon Lohman, Suraj Suresh, Abdullah Naji, Sarah Russell, Eva Alsheik, Keith Mullins

**Affiliations:** 1 Internal Medicine, Northwestern University Feinberg School of Medicine, Chicago, USA; 2 Department of Internal Medicine, University of Michigan, Ann Arbor, USA; 3 Division of Gastroenterology and Hepatology, Henry Ford Health System, Detroit, USA; 4 Department of Anaesthesiology, Oregon Health & Science University, Portland, USA

**Keywords:** pelvic floor dysfunction, constipation, anorectal manometry, peripheral neuropathy, autoimmune autonomic ganglionopathy

## Abstract

Autoimmune autonomic ganglionopathy (AAG) is a rare post-ganglionic disorder that causes a range of symptoms, often including gastrointestinal disorders. Patients may be seropositive or seronegative for antibodies against the nicotinic acetylcholine receptor. Here, we describe the case of a 56-year-old woman with a previous diagnosis of sensorimotor peripheral neuropathy who presented with severe constipation that was not responsive to laxative therapy. The evaluation showed diffuse colonic hypomotility, rectal hypersensitivity, and type IV pelvic floor dysfunction. The patient was diagnosed 10 months after the presentation as having seronegative AAG, and she responded well to treatment with intravenous methylprednisolone and apheresis.

## Introduction

Autoimmune autonomic ganglionopathy (AAG) is a rare disease with various manifestations. AAG is primarily a post-ganglionic disorder thought to involve antibodies that target the nicotinic acetylcholine receptor (gAChR) [1]. In the idiopathic form, it is thought that the body spontaneously generates autoantibodies against gAChR [2]. When caused by a primary neoplasm, the immune system produces paraneoplastic antibodies that cross-react and bind to the body’s gAChR system [3].

Most patients with AAG experience some form of gastrointestinal (GI) dysmotility alongside other manifestations of sympathetic and parasympathetic dysfunction [4]. Furthermore, in the idiopathic form, seronegative and seropositive forms of AAG have been identified. Here, we present the case of a patient with seronegative AAG who was diagnosed 10 months after an initial presentation to the general GI clinic. The patient initially presented with severe constipation and eventually showed clinical improvement after target therapy was started.

This article was previously presented as a meeting abstract at the 2021 American College of Gastroenterology national meeting held on October 24, 2021.

## Case presentation

A 56-year-old woman with a medical history of idiopathic sensorimotor peripheral neuropathy, fibromyalgia, anxiety, depression, and schwannomatosis presented to the gastroenterology clinic with severe constipation. She had no significant surgical history (including abdominal and neurological surgery), and her family history was significant for neuropathy in her brother and colorectal cancer in two aunts. Her severe constipation was associated with weakness, nausea, weight loss, and urinary symptoms, including urinary and defecation urgency and straining for approximately six weeks. The patient’s bowel habits had decreased from one bowel movement per day to one every fourth day. Prior to the presentation, the patient had tried enemas, magnesium citrate, and bisacodyl unsuccessfully.

**Figure 1:**
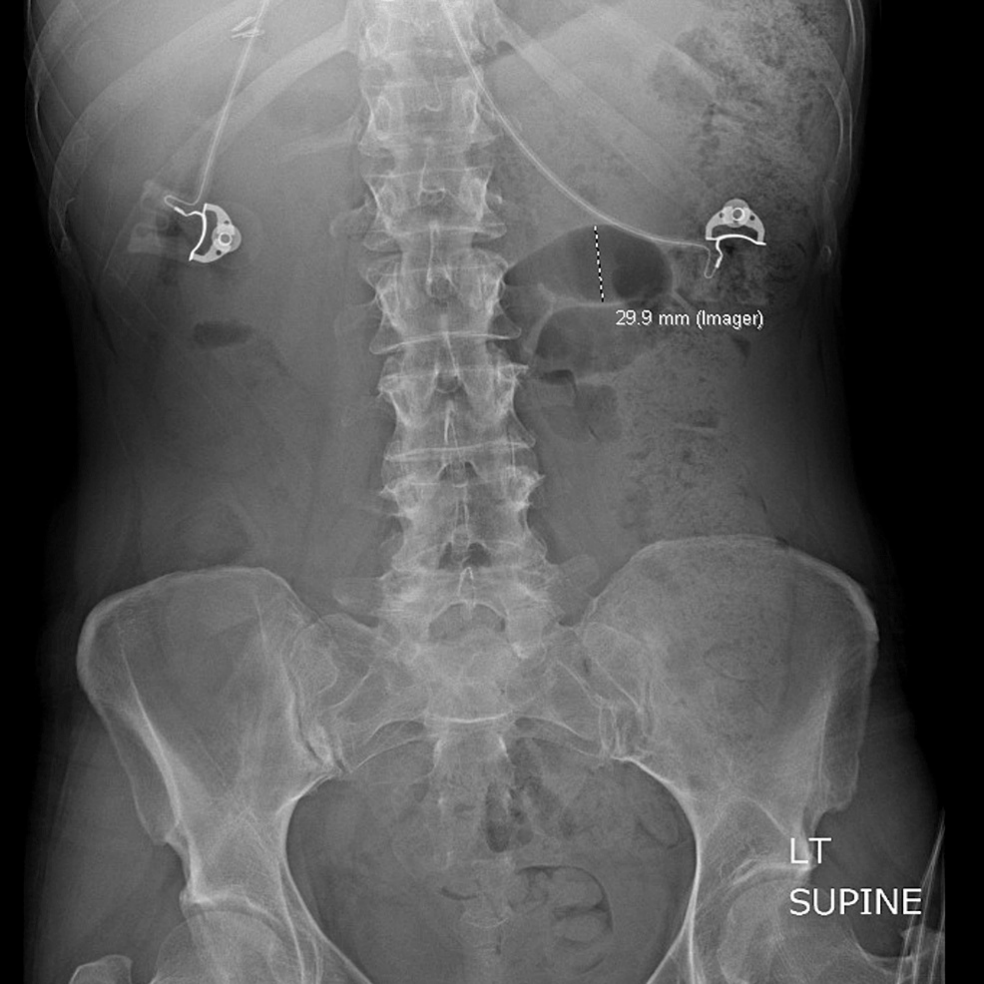
Abdominal X-ray revealing a focal dilated gas-filled loop of the small bowel in the left hemiabdomen measuring up to 3.0 cm, possibly reflecting focal ileus.

The patient was referred to the general GI clinic after multiple visits to the emergency department (ED) for the abovementioned symptoms. In the ED, the patient was found to have orthostatic hypotension. All other vital signs were normal. An abdominal X-ray performed at the time was concerning for focal ileus (Figure 1). Given her family history of colorectal cancer, the patient was referred for colonoscopy, which was unremarkable. Laxative medications were not effective, prompting a colonic transit study and anorectal manometry.

**Figure 2:**
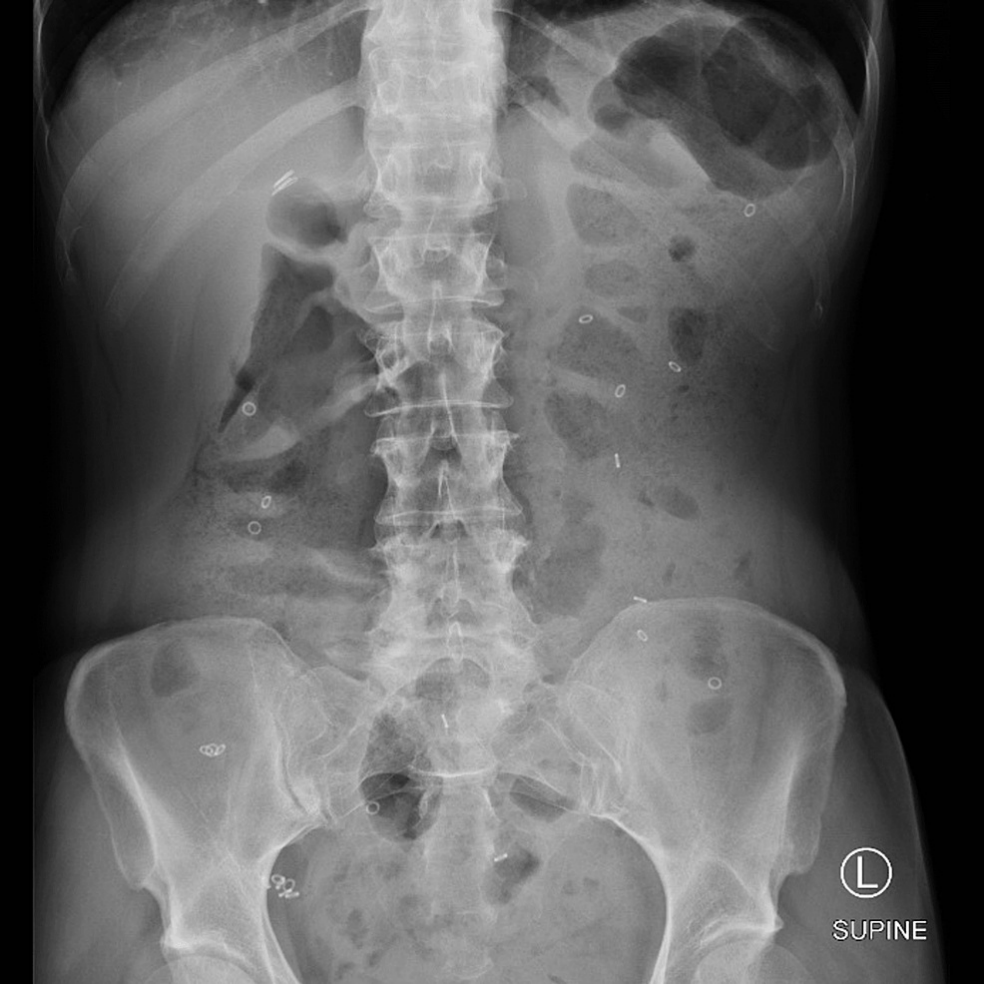
Colonic transit study displaying 24 Sitzmarks present throughout the entire colon uniformly indicative of diffuse colonic hypomotility/inertia.

**Figure 3:**
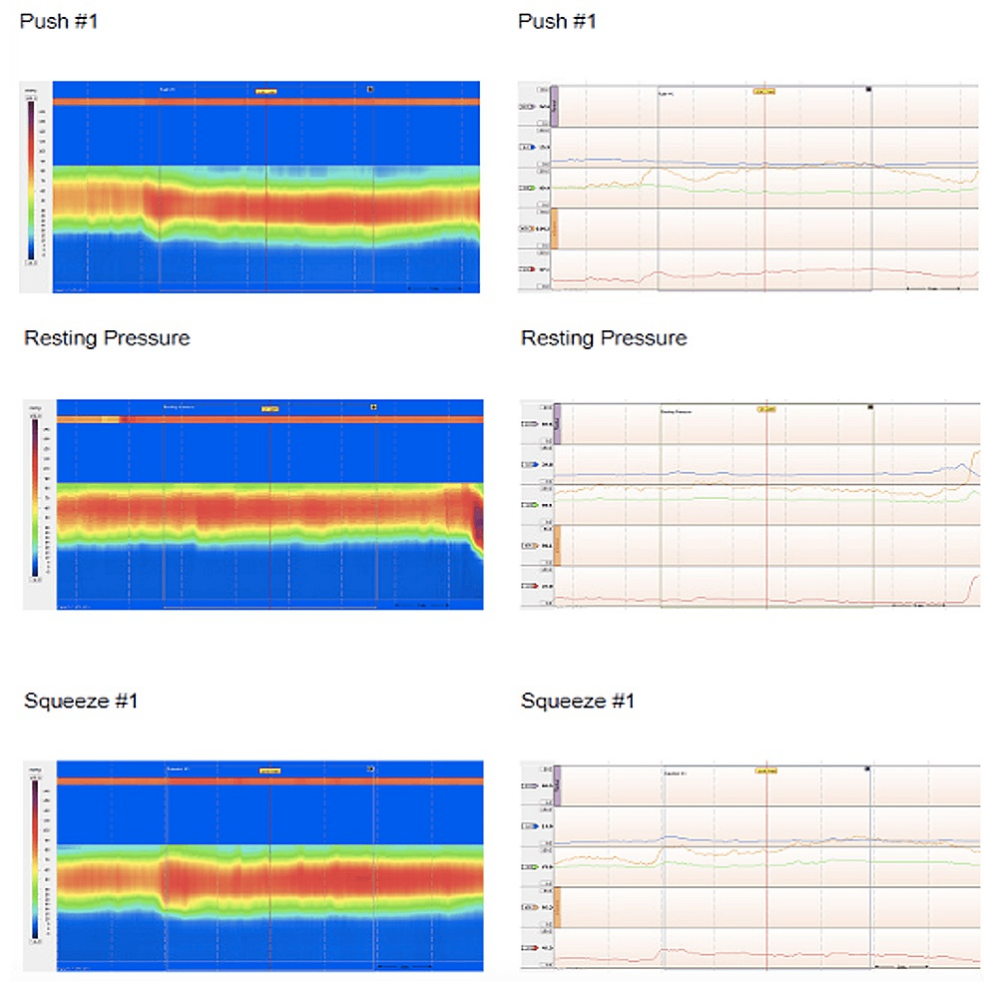
Anorectal manometry. The overall basal resting anal sphincter pressure was elevated at 97.2 mmHg. Overall maximum squeeze pressure was 106.9 mmHg, which is only a slight increase from baseline pressure. During bear down exercises, the rectal pressure did not increase appropriately from baseline, and the anal sphincter incompletely relaxed during simulated defecation.

The colonic transit study revealed diffuse colonic hypomotility/inertia (Figure 2). Anorectal manometry was significant for rectal hypersensitivity including elevated baseline anal sphincter pressure (97.2 mmHg) and anal sphincter pressure upon maximum squeeze (16.8 mmHg) and bear down exercises (89.9 mmHg), as well as type IV pelvic floor dysfunction (Figure 3). The rectoanal inhibitory reflex was present. Minimal variation in anal sphincter pressures was noted between exercises (i.e., rest, squeeze, and bear down). The patient’s pelvic floor dysfunction did not respond to physical therapy. This led to a referral to a motility specialist and a second opinion from a neurologist at a quaternary care center. The patient was then given an autonomic reflex screen test, which is a noninvasive test used to assess how the nervous system works to control blood pressure, heart rate, and sweating. The patient’s autonomic reflex screen was positive. Her anti-gAChR antibody assay was negative.

At 10 months after her initial clinic presentation, the patient was given the diagnosis of seronegative AAG. Although the patient had a negative anti-gAChR antibody assay, her broad symptoms and positive autonomic reflex screen test favored a diagnosis of AAG over her previous diagnosis of sensorimotor peripheral neuropathy. The treatment plan included intravenous (IV) methylprednisolone 1 g weekly for 12 weeks and three months of apheresis based on expert opinion [5]. At the time of writing, no treatment protocol has been standardized for the treatment of AAG given its rarity. Upon treatment completion, the patient’s symptoms had improved.

## Discussion

AAG is a rare disorder of the autonomic nervous system caused by antibodies against gAChR, resulting in failure of the sympathetic, parasympathetic, and enteric systems. Middle-aged women are most frequently diagnosed with AAG [6]. Approximately half of all AAG patients are found to have antibodies against gAChR [6]. For patients who are seronegative for anti-gAChR antibodies, it is hypothesized that other antibodies may cause autonomic dysfunction. Our patient presented with several weeks of worsening constipation and other autonomic symptoms and was found to have negative gAChR serology. Her symptoms were attributed to her past diagnosis of sensorimotor peripheral neuropathy. It took 10 months for the correct clinical diagnosis to be made due to the complexity of AAG. Because AAG presents with various symptoms, physicians must understand its clinical presentation.

AAG has multisystem effects disrupting cardiovascular, gastrointestinal, urogenital, thermoregulatory, sudomotor, and pupillomotor functions [7]. Therefore, a strong multidisciplinary team is necessary for appropriate diagnosis. In a cohort study of 179 seropositive patients with AAG, the most frequent symptoms were orthostatic hypotension (134/179), orthostatic intolerance (148/179), and lower GI dysfunction such as constipation and diarrhea (132/179) [8]. However, limited studies exist describing patients with seronegative AAG. In one case series of six seronegative patients, presenting symptoms included paresthesia, GI symptoms, and orthostatic hypotension, and as the disease progressed, orthostatic hypotension, GI symptoms, and urinary retention became more prominent [9]. AAG causes GI symptoms by disrupting the enteric system that controls GI behavior by interacting with the gut’s immune and endocrine systems, regulating motility, gastric acid secretion, fluid and nutrient movement, and blood flow [10]. GI tract symptoms are found in 83-91.7% of patients with seropositive AAG [11] and include upper GI symptoms, lower GI symptoms of constipation and diarrhea, and motility issues. In patients with a history of autoimmune and autonomic disorders, constipation should alert a physician to keep AAG within the differential diagnosis as a possible disease etiology.

Antibody-targeted therapies such as intravenous immunoglobulin (IVIG) and plasma exchange (PLEX) are considered first-line treatments for AAG. Interestingly, Golden et al. observed that patients with negative serology responded best to steroids rather than IVIG, PLEX, or rituximab [9]. 

## Conclusions

Our patient’s symptoms improved with a combination of IV methylprednisolone and apheresis after correct diagnosis. Recognizing AAG in the differential diagnosis for patients with GI dysfunction associated with autonomic symptoms is critical, especially because effective treatment options exist.
